# Lung Cancer Disparities in the United States: The Role of Smoking, Comorbidities, Socioeconomic Status, and Regional Variation

**DOI:** 10.7759/cureus.110266

**Published:** 2026-06-04

**Authors:** Bugra Zengin, Canan D Dirican, Salih Akgun, Jamil Nazzal, Mohammad Salameh, Jasneet Randhawa, Nimrat Bains, Lisa Duhaime, Sanjay Jain

**Affiliations:** 1 Internal Medicine, Hamilton Medical Center, Dalton, USA; 2 Internal Medicine, New York Medical College Internal Medicine Residency Program at St. Mary's General Hospital and Saint Clare’s Hospitals, Denville, USA; 3 Internal Medicine, John F. Kennedy University Medical Center, Edison, USA; 4 Internal Medicine, Vanderbilt University Medical Center, Nashville, USA; 5 Hematology and Medical Oncology, Hamilton Medical Center, Dalton, USA; 6 Hematology and Medical Oncology, Medical University of South Carolina (MUSC) Hollings Cancer Center, Charleston, USA

**Keywords:** comorbidities, disparities, lung cancer, regional variation, socioeconomic factors

## Abstract

Background

Lung cancer disproportionately affects certain racial and regional populations in the United States, with notable disparities observed among patients hospitalized with this diagnosis. This study examines disparities in clinical characteristics, socioeconomic distribution, and hospital outcomes among adults hospitalized with lung cancer.

Methods

A retrospective analysis was performed using the 2019-2020 National Inpatient Sample (NIS). Patients with a primary diagnosis of lung cancer were evaluated across racial groups and U.S. regions. Key variables included smoking status, comorbidity burden, length of stay (LOS), in-hospital mortality, and total hospital charges.

Results

White patients demonstrated the highest prevalence of smoking, whereas Black patients experienced the longest LOS. Hispanic patients had the highest total hospital charges. Regionally, lung cancer admissions were most common in the South, which also showed lower socioeconomic status and reduced access to screening. In unadjusted analyses, in-hospital mortality did not differ significantly by hospital region; however, after multivariable adjustment, patients hospitalized in the South had significantly lower odds of in-hospital mortality compared with those hospitalized in the Northeast. Socioeconomic status showed a graded association, with progressively lower mortality risk among patients in higher income quartiles.

Conclusions

Significant racial and regional disparities exist in lung cancer burden and hospital outcomes. Targeted interventions addressing socioeconomic barriers, screening inequities, and comorbidity management are needed to reduce these disparities.

## Introduction

Lung cancer remains the leading cause of cancer-related mortality in the United States, accounting for approximately 125,000 deaths annually [1]. Although lung cancer incidence and mortality have declined over recent decades, largely driven by reductions in smoking and advances in early detection and treatment, overall survival remains poor, with a five-year survival rate of approximately 20% [1-3]. Prognosis is influenced by multiple factors, including tumor stage and histology, comorbidity burden, behavioral risk factors such as smoking, and access to timely, guideline-concordant care [4,5]. Substantial disparities in lung cancer outcomes have been consistently documented across racial, ethnic, socioeconomic, and geographic groups [6]. Differences in survival by race and ethnicity are not fully explained by biological factors or tumor genomics, as studies evaluating mutation frequencies and ancestry-related markers have not identified consistent biological drivers of these disparities [7,8]. Instead, a growing body of evidence suggests that inequities in access to care, quality of treatment, and timeliness of diagnosis, often shaped by socioeconomic disadvantage, play a central role in determining stage at presentation and subsequent outcomes [9,10]. Comorbidity burden further complicates this relationship, as chronic conditions that disproportionately affect minority populations may limit treatment options and worsen survival [5,11].

Geographic variation also contributes meaningfully to lung cancer burden and outcomes. States in the South consistently demonstrate higher lung cancer incidence and mortality, a pattern associated with socioeconomic disadvantage, lower access to preventive services, and reduced uptake of lung cancer screening [1,9,12]. In contrast, Western regions often exhibit lower incidence but higher hospital charges and distinct patterns of resource utilization, reflecting regional differences in healthcare delivery and pricing structures [13]. These geographic and socioeconomic factors intersect with race and ethnicity to shape patterns of disease presentation, hospitalization, and outcomes.

Despite extensive literature on lung cancer disparities, most studies have focused on incidence, stage at diagnosis, or long-term survival, with less attention given to disparities in inpatient outcomes and healthcare utilization during hospitalization [14]. Moreover, disparities are often examined in isolation, without accounting for the intersection of race, socioeconomic status, comorbidity burden, and geographic region. To address these gaps, we used a nationally representative inpatient database to evaluate racial, socioeconomic, and regional disparities in lung cancer hospitalizations, focusing on in-hospital mortality, length of stay (LOS), and total hospital charges. By examining these intersecting factors, our study aims to provide a more granular understanding of structural inequities that may shape inpatient lung cancer care in the United States.

This article was previously posted as a preprint on Research Square on March 08, 2026.

## Materials and methods

We conducted a cross-sectional observational study using the 2019-2020 National Inpatient Sample (NIS), the largest publicly available all-payer inpatient database in the United States. Adults aged ≥18 years with a principal diagnosis of lung cancer were identified using ICD-10-CM codes (C34.00, C34.01, C34.02, C34.10, C34.11, C34.12, C34.2, C34.30, C34.31, C34.32, C34.80, C34.81, C34.82, C34.90, C34.91, C34.92) previously validated for epidemiologic surveillance [13]. These codes collectively represent malignant neoplasms of the bronchus and lung across the anatomic sites captured by ICD-10-CM category C34, including tumors of the main bronchus, upper, middle, and lower lobes, overlapping sites, and unspecified sites of the bronchus and lung, consistent with standard ICD-10-CM classification for epidemiologic surveillance. Smoking status and comorbidities, including chronic obstructive pulmonary disease (COPD), congestive heart failure, diabetes mellitus, chronic kidney disease, and obesity, were identified using their respective ICD-10-CM diagnosis codes present in the inpatient record. We quantified regional admission prevalence by calculating the survey-weighted proportion of hospitalizations attributed to a principal diagnosis of lung cancer across the four U.S. Census hospital regions: Northeast, Midwest, South, and West. Patients were further stratified by race/ethnicity (White, Black, Hispanic, Other), with the Other category representing Asian or Pacific Islander, Native American, or individuals classified as Other in the NIS dataset.

Primary study variables of interest included LOS, in-hospital mortality, total hospital charges, smoking prevalence, and distribution across income quartiles. Secondary analyses compared regional patterns of lung cancer admission prevalence and regional smoking prevalence, reflecting known geographic variation in lung cancer risk and healthcare access across the United States. Accordingly, observed disparities reflect differences in the inpatient burden of care rather than disease incidence or prevalence.

All analyses were performed using Stata/SE 17.0 (StataCorp LLC, College Station, TX). Given the complex survey design of the NIS, we applied survey weights, strata, and cluster variables in accordance with Healthcare Cost and Utilization Project (HCUP) recommendations. Descriptive statistics were compared using chi-square tests for categorical variables and ANOVA for continuous variables. Multivariable models incorporated survey-weighted procedures to preserve national representativeness. Statistical significance was defined as p < 0.05.

Multivariable survey-weighted logistic and linear regression models were constructed to evaluate the independent associations of race with in-hospital mortality, LOS, and total hospital charges. Race was modeled as a categorical variable, with White patients serving as the reference group, and all models were adjusted for age, sex, smoking status, comorbidities (COPD, congestive heart failure, diabetes mellitus, chronic kidney disease, and obesity), income quartile, and hospital region.

This study used publicly available, de-identified data and was therefore exempt from Institutional Review Board approval.

## Results

Patient characteristics by race

A total of 213,435 weighted hospitalizations for primary lung cancer were included in the analysis. The mean age of the cohort was 68.8 years, and 50.5% (n = 107,763) of hospitalizations were among female patients. The racial distribution of hospitalizations was approximately 75% White (n = 159,158), 12% Black (n = 26,338), 5% Hispanic (n = 10,160), and 8% classified as Other (n = 17,715). Minor discrepancies between summed subgroup counts and the total cohort are attributable to survey-weighted rounding across independent cross-tabulations, consistent with the NIS complex survey design.

Significant racial differences were observed in baseline demographic and clinical characteristics. The proportion of patients older than 60 years was highest among White patients (n = 134,027; 84.21%). Documented smoking prevalence differed significantly across racial groups (p < 0.001), with White patients having the highest proportion of smokers (n = 75,520; 47.45%), followed by Hispanic patients (n = 4,230; 41.63%), Black patients (n = 10,688; 40.58%), and patients categorized as Other race (n = 6,813; 38.46%).

Comorbidity prevalence also varied by race. COPD was most frequent among White patients (n = 62,549; 39.30%), followed by Black patients (n = 9,042; 34.33%) and Hispanic patients (n = 2,673; 26.31%). Congestive heart failure was most prevalent among Black patients (n = 3,798; 14.42%), compared with White patients (n = 19,115; 12.01%) and Hispanic patients (n = 1,127; 11.09%). Diabetes mellitus was most common among Hispanic patients (n = 3,158; 31.08%). Obesity was most prevalent among White patients (n = 19,051; 11.97%) and least prevalent among patients classified as Other race (n = 1,175; 6.63%). Chronic kidney disease was most prevalent among Black patients (n = 4,369; 16.59%). Detailed baseline characteristics by race are presented in Table 1.

**Table 1:** Racial differences in clinical characteristics. COPD: Chronic obstructive pulmonary disease; CHF: Congestive heart failure; CKD: Chronic kidney disease; n: Number of patients.

Variable	White, n (%)	Black, n (%)	Hispanic, n (%)	Other, n (%)	p-value
Smoking	75,520 (47.45%)	10,688 (40.58%)	4,230 (41.63%)	6,813 (38.46%)	<0.001
COPD	62,549 (39.30%)	9,042 (34.33%)	2,673 (26.31%)	4,716 (26.62%)	<0.001
CHF	19,115 (12.01%)	3,798 (14.42%)	1,127 (11.09%)	1,741 (9.83%)	<0.001
Diabetes	32,787 (20.60%)	7,346 (27.89%)	3,158 (31.08%)	4,606 (26.00%)	<0.001
Obesity	19,051 (11.97%)	3,032 (11.51%)	1,137 (11.19%)	1,175 (6.63%)	<0.001
CKD	18,685 (11.74%)	4,369 (16.59%)	1,247 (12.27%)	1,791 (10.11%)	<0.001
Age >60 years	134,027 (84.21%)	20,267 (76.95%)	8,030 (79.04%)	13,993 (78.99%)	<0.001

Hospital outcomes by race

Mean LOS differed significantly across racial groups (p < 0.001). Black patients had the longest mean LOS (6.95 days), followed by Hispanic patients (6.62 days), patients categorized as Other race (6.27 days), and White patients (5.83 days). Regression analysis confirmed an association between race and LOS (β = 0.262, p < 0.001). In-hospital mortality also differed significantly by race (p = 0.0005). Patients categorized as Other race had the highest in-hospital mortality rate at 7.88% (n = 1,396), followed by Hispanic patients at 7.41% (n = 753), Black patients at 7.22% (n = 1,902), and White patients at 6.29% (n = 10,011).

In-hospital mortality rates by race are reported later as part of the multivariable analysis. Total hospital charges also varied: Hispanic patients had the highest mean charges ($114,594), followed by patients categorized as Other race ($102,646), Black patients ($88,498), and White patients ($87,058). Mean LOS and total hospital charges by racial group are summarized in Table 2.

**Table 2:** Hospital outcomes by race. LOS: Length of stay; n: Number of patients.

Outcome	White (n = 159,158)	Black (n = 26,338)	Hispanic (n = 10,160)	Other (n = 17,715)
Mean LOS (days)	5.83	6.95	6.62	6.27
In-hospital mortality, n (%)	10,011 (6.29%)	1,902 (7.22%)	753 (7.41%)	1,396 (7.88%)
Mean total hospital charges ($)	87,058	88,498	114,594	102,646

Regional variation

Regional analyses demonstrated substantial geographic disparities in lung cancer hospitalizations. The South accounted for the highest proportion of admissions at 39.5% (n = 84,307), followed by the Midwest (n = 49,645; 23.3%), Northeast (n = 46,870; 22.0%), and West (n = 32,656; 15.3%). Documented smoking prevalence also differed significantly across regions (p < 0.001). The Northeast demonstrated the highest proportion of smokers at 48.4%, followed by the Midwest at 47.0% and the West at 46.6%, while the South had the lowest smoking prevalence at 42.9%.

Race distributions varied significantly by hospital region (p < 0.001). Hospitalizations among White patients were most heavily concentrated in the South, representing 38.6% (n = 61,435) of all White lung cancer hospitalizations, while hospitalizations among Black patients were even more concentrated in this region at 53.8% (n = 14,170). Hispanic patients showed a different pattern, with the West accounting for 29.1% (n = 2,957) of all Hispanic lung cancer hospitalizations and the Northeast accounting for 21.9% (n = 2,225). Hospitalizations among patients categorized as Other race were most concentrated in the West at 32.4% (n = 5,740), followed by the Northeast at 24.6% (n = 4,358).

Income patterns also demonstrated regional variation. The lowest national income quartile accounted for 28.6% of all lung cancer hospitalizations nationwide and was disproportionately represented in the South, where 40.26% (n = 33,925) of patients were from the lowest-income areas. The Midwest also showed elevated representation at 27.25% (n = 13,528). In contrast, the Northeast and West had lower proportions from the lowest-income quartile at 17.25% (n = 8,085) and 17.08% (n = 5,578), respectively. Patients from the highest income quartile were most concentrated in the Northeast (n = 16,433; 35.06%) and West (n = 10,071; 30.84%) and least represented in the South (n = 10,162; 12.06%).

**Figure 1:**
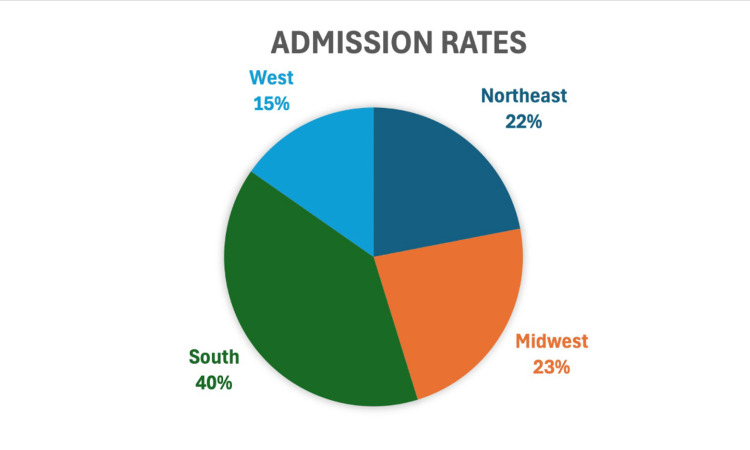
Distribution of clinical and regional characteristics.

In unadjusted analyses, inpatient mortality did not differ significantly across regions (p = 0.185); however, multivariable adjusted analyses revealed that patients hospitalized in the South had significantly lower odds of in-hospital mortality compared with the Northeast (aOR 0.76, 95% CI 0.62-0.93, p = 0.008). Regional and racial distributions of clinical characteristics are summarized in Figure 1. Regional variation in smoking prevalence, mortality, and income distribution is detailed in Table 3.

**Table 3:** Regional variation in smoking prevalence, in-hospital mortality, and income distribution. n: Number of patients; Q1: Lowest income quartile; Q4: Highest income quartile. P-values: smoking, p < 0.001; in-hospital mortality, p = 0.185; Q1 income quartile, p < 0.001; Q4 income quartile, p < 0.001.

Region	Smoking, n (%)	In-hospital mortality, n (%)	Q1 income quartile, n (%)	Q4 income quartile, n (%)
Northeast	22,685 (48.4%)	3,440 (7.34%)	8,085 (17.25%)	16,433 (35.06%)
Midwest	23,333 (47.0%)	3,182 (6.41%)	13,528 (27.25%)	7,114 (14.33%)
South	36,149 (42.9%)	5,283 (6.27%)	33,925 (40.26%)	10,162 (12.06%)
West	15,218 (46.6%)	2,165 (6.63%)	5,578 (17.08%)	10,071 (30.84%)

Socioeconomic status and racial distribution

Income quartile demonstrated a strong association with race (p < 0.001). Black patients had the highest concentration in the lowest national income quartile, with 55.05% (n = 14,499) residing in ZIP codes corresponding to the lowest 25% of median household income. Hispanic patients also showed a high proportion in this quartile, at 38.21% (n = 3,882). In contrast, White patients were more evenly distributed across income levels, with 24.49% (n = 38,978) in the lowest quartile and a substantially higher proportion in the highest income quartile. Patients categorized as Other race similarly showed greater representation in the upper income quartiles compared with Black and Hispanic patients. Income quartile distributions by race are shown in Table 4.

**Table 4:** Income quartile distribution by race. Income quartile analyses were limited to hospitalizations with available ZIP code income data; therefore, totals may differ from the full cohort.

Income quartile	White, n (%)	Black, n (%)	Hispanic, n (%)	Other, n (%)	p-value
Q1 (lowest)	38,978 (24.49%)	14,499 (55.05%)	3,882 (38.21%)	3,747 (21.15%)	<0.001
Q2	44,898 (28.21%)	5,618 (21.33%)	2,473 (24.34%)	4,037 (22.79%)	
Q3	40,824 (25.65%)	3,793 (14.40%)	2,279 (22.43%)	4,572 (25.81%)	
Q4 (highest)	34,442 (21.64%)	2,431 (9.23%)	1,526 (15.02%)	5,359 (30.25%)	

Multivariable analyses of mortality, LOS, and hospital charges

In survey-weighted multivariable logistic regression adjusting for sex, age, smoking status, comorbidities (COPD, CHF, diabetes mellitus, chronic kidney disease, and obesity), income quartile, and hospital region, race remained independently associated with in-hospital mortality. Compared with White patients, who served as the reference group, Black patients had significantly higher odds of in-hospital mortality (adjusted OR 1.10, 95% CI 1.04-1.17, p = 0.002), as further detailed in Table 5. Female sex was associated with significantly lower odds of in-hospital mortality (aOR 0.77, 95% CI 0.71-0.83, p < 0.001). COPD (aOR 1.21), CHF (aOR 1.69), and CKD (aOR 1.20) were independently associated with increased odds of mortality, while smoking status and obesity were associated with lower odds of in-hospital mortality. Income quartile demonstrated a graded inverse association, with patients from higher-income ZIP codes experiencing lower odds of mortality compared with those from the lowest-income quartile.

**Table 5:** Multivariable survey-weighted logistic regression for in-hospital mortality. Outcome: In-hospital mortality Reference groups: White race, male sex, income quartile 1 (lowest), and hospital region 1 (Northeast). Hospital regions: 1 = Northeast, 2 = Midwest, 3 = South, and 4 = West.

Variable	Adjusted OR	95% CI	p-value
Race			
Black vs. White	1.1	1.04-1.17	0.002
Hispanic vs. White	1.05	0.96-1.15	0.278
Other vs. White	1.03	0.95-1.12	0.462
Female sex	0.77	0.71-0.83	<0.001
COPD	1.21	1.11-1.31	<0.001
Congestive heart failure	1.69	1.52-1.89	<0.001
Diabetes mellitus	0.94	0.85-1.03	0.182
Chronic kidney disease	1.2	1.07-1.35	0.002
Smoking	0.65	0.60-0.71	<0.001
Age >60 years	1.01	0.91-1.12	0.85
Obesity	0.68	0.59-0.79	<0.001
Income quartile 2 vs. 1	0.89	0.81-0.99	0.026
Income quartile 3 vs. 1	0.82	0.74-0.91	<0.001
Income quartile 4 vs. 1	0.78	0.69-0.88	<0.001
Midwest vs. Northeast	0.82	0.67-1.00	0.056
South vs. Northeast	0.76	0.62-0.93	0.008
West vs. Northeast	0.87	0.70-1.09	0.224

In survey-weighted multivariable linear regression, race remained an independent predictor of LOS. After full adjustment, Black race, compared with White race, was independently associated with longer hospitalization (β = 0.26 days, 95% CI 0.18-0.34, p < 0.001), with White patients serving as the reference group. Female sex was associated with shorter LOS, while COPD, CHF, and CKD were associated with significantly longer hospitalizations. Increasing income quartile was associated with progressively shorter LOS, suggesting a socioeconomic gradient in inpatient utilization. Regional differences persisted, with shorter LOS observed in the Northeast and West compared with the reference region.

In adjusted analyses of total hospital charges, race remained significantly associated with increased charges (β = $4,458, 95% CI $2,933-$5,984, p < 0.001), independent of demographic, clinical, socioeconomic, and regional factors. Female sex and smoking status were associated with lower charges, while COPD, CHF, and obesity were associated with significantly higher hospital charges. Patients treated in Western hospitals incurred substantially higher charges, whereas treatment in hospitals in the Northeast was associated with lower charges, reflecting persistent regional variation in healthcare pricing.

## Discussion

This nationally representative analysis reveals substantial racial, regional, and socioeconomic disparities among hospitalized lung cancer patients in the United States.

Racial variation in smoking and comorbidity burden

Prior research has shown that racial disparities in lung cancer outcomes persist even after accounting for smoking exposure, suggesting that differences in comorbidity burden, disease severity, and access to care play important roles [5,7]. Black and Hispanic patients have been reported to experience worse clinical outcomes despite similar or lower smoking prevalence compared with White patients [15,16]. Consistent with this literature, we found that although White patients had the highest smoking prevalence, Black and Hispanic patients experienced worse inpatient outcomes, including longer lengths of stay and greater healthcare utilization, indicating that smoking behavior alone does not explain the observed disparities.

Although patients aged 60 years or older comprised more than 75% of hospitalizations across all racial groups, the clinical meaningfulness of this finding is limited given the similar age distribution observed across groups. The statistical significance likely reflects the overall demographic profile of lung cancer as a disease of older adults rather than a meaningful racial difference in age at hospitalization. It should also be noted that the current study is limited to administrative inpatient data and does not include genomic or genetic information. Future studies integrating molecular and genomic data with administrative claims could help disentangle biological from structural contributors to racial disparities in lung cancer outcomes.

Ryan BM also described earlier lung cancer onset among racial and ethnic minority populations, attributed to socioeconomic disadvantage, cumulative environmental exposures, delayed screening, and a higher burden of systemic risk factors [16]. In our cohort, Black and Hispanic patients were younger at hospitalization than White patients, a pattern that may reflect differences in disease burden and access to earlier detection. The literature further demonstrates that Black and Hispanic populations bear a disproportionate burden of chronic comorbidities such as congestive heart failure, chronic kidney disease, and diabetes, reflecting long-standing inequities in preventive care [11,17,18]. We similarly observed higher prevalence of these conditions among Black and Hispanic patients, which likely contributed to prolonged hospitalization and increased resource utilization.

Importantly, prior work by Soneji S et al. suggests that racial disparities in lung cancer outcomes often persist after adjustment for clinical factors [19]. In our fully adjusted models accounting for smoking status, comorbidity burden, socioeconomic status, and geographic region, race remained independently associated with longer LOS, higher hospital charges, and modestly increased odds of in-hospital mortality, highlighting the role of broader structural and systemic factors in shaping inpatient lung cancer outcomes.

Resource utilization differences

Prior studies have shown that racial and ethnic differences in hospital resource utilization reflect not only disease severity but also structural factors such as access to outpatient care, insurance coverage, and regional variation in healthcare pricing. Black patients, in particular, have been reported to experience longer hospitalizations, often attributed to delayed presentation, higher comorbidity burden, and barriers to timely ambulatory care [13,20].

Consistent with this literature, we found that Black patients had the longest lengths of stay. This pattern likely reflects a greater burden of chronic comorbidities and more complex inpatient management needs and may also be influenced by limited access to preventive and outpatient care, although such mechanisms cannot be directly assessed in an inpatient-only dataset.

Hospital charges demonstrated a different pattern, with Hispanic patients incurring the highest total hospital charges despite shorter average lengths of stay. While Hispanic patients were frequently hospitalized in the South, they were also disproportionately represented in the West and Northeast, regions known to have substantially higher healthcare charges compared with the South and Midwest. Regional pricing variation alone may therefore account for a meaningful portion of the observed charge differences [13].

Beyond geography, prior studies suggest that differences in diagnostic intensity, procedural utilization, and patterns of care-seeking once hospitalized may contribute to increased inpatient charges among Hispanic patients, even in the absence of prolonged hospitalization [21,22]. Socioeconomic constraints, including underinsurance and limited access to timely outpatient evaluation, may further lead to more resource-intensive inpatient care.

In adjusted analyses, racial differences in total hospital charges persisted after accounting for LOS, comorbidity burden, income quartile, and hospital region, indicating that excess charges are not solely driven by longer hospitalization or measurable clinical severity and likely reflect a combination of regional pricing structures and structural barriers to outpatient care.

Geographic disparities

Prior research has shown that the Southern United States bears a disproportionate burden of lung cancer, driven by socioeconomic disadvantage, limited access to preventive care, and structural barriers to early detection. Some studies have also reported lower documented smoking prevalence in the South, a finding often attributed to underreporting, inconsistent documentation, and reduced engagement in preventive healthcare rather than true differences in exposure.

Consistent with this literature, we found that the South accounted for nearly two-fifths of all lung cancer hospitalizations despite having the lowest recorded smoking prevalence in our cohort. This apparent discrepancy likely reflects incomplete capture of smoking behavior as well as broader gaps in risk awareness and routine healthcare utilization [23].

Limited access to lung cancer screening may further contribute to these patterns. Southern states have among the lowest rates of low-dose CT screening nationally despite high screening eligibility, and reduced screening has been associated with later-stage diagnosis and increased reliance on inpatient care [4,12,24]. Our findings of a higher hospitalization burden in the South align with these observations.

Socioeconomic disadvantage appears to amplify regional differences. The South had the highest proportion of patients residing in the lowest income quartile, a factor linked to delayed presentation and increased dependence on hospital-based care. Although inpatient mortality did not differ significantly by region, disparities in stage at diagnosis and access to definitive treatment may influence outcomes beyond hospitalization.

Finally, regional variation in resource utilization persisted after adjustment, with hospitals in the West incurring higher total charges and those in the Northeast demonstrating shorter stays and lower charges. These differences likely reflect regional pricing and practice patterns rather than disease biology alone.

Intersection of race and socioeconomic status

Prior studies have consistently demonstrated pronounced socioeconomic gradients across racial and ethnic groups, with Black and Hispanic populations disproportionately residing in lower-income neighborhoods. These structural inequities influence housing stability, educational and employment opportunities, environmental exposures, insurance coverage, and access to preventive healthcare [25]. Lower neighborhood income has been closely linked to reduced primary care access, fewer preventive services, and limited availability of accredited lung cancer screening programs, contributing to delayed diagnosis and more advanced disease at presentation [26].

Consistent with this literature, we observed that Black patients were most heavily represented in the lowest income quartile, followed by Hispanic patients, whereas White and Other race patients were more frequently concentrated in higher income quartiles. Among Black patients, historical and systemic factors, including residential segregation, disproportionate environmental exposures, and long-standing barriers to wealth accumulation, likely contribute to reduced engagement in preventive care and delayed evaluation of respiratory symptoms, resulting in higher inpatient utilization [23,25,27].

Hispanic patients similarly demonstrated a substantial concentration in lower income quartiles, reflecting additional challenges related to immigration status, language barriers, underinsurance, and employment in sectors with limited health benefits [28-30]. These barriers may limit access to routine care and screening awareness, leading to more advanced disease or more resource-intensive inpatient evaluations, which may help explain the higher hospital charges observed in this group.

Overall, the intersection of race and socioeconomic status plays a central role in shaping lung cancer presentation, screening uptake, and inpatient resource utilization. Our findings reinforce the need for targeted strategies to expand equitable screening access, strengthen culturally informed preventive care, and address upstream socioeconomic determinants that contribute to persistent disparities in lung cancer outcomes.

Policy and practice implications

Targeted strategies are needed to reduce disparities in lung cancer outcomes. Expanding access to low-dose CT screening, particularly in regions and populations with low uptake, may help facilitate earlier detection. Improving access to primary care and strengthening chronic disease management could reduce disease severity at presentation and decrease reliance on inpatient care. Policies that address structural determinants of health, including insurance coverage, transportation barriers, and regional shortages of specialty services, are also essential. In addition, culturally tailored smoking cessation programs and community-based education efforts may help engage high-risk groups and reduce preventable disease burden. A visual overview of the key findings is provided in the graphical abstract.

Strengths and limitations

Strengths include the use of a large, nationally representative database and robust survey-weighted analyses. Limitations include the inability to assess cancer stage, outpatient care patterns, time to treatment, or tumor biology, all of which influence outcomes. The NIS reflects inpatient hospitalization data and does not capture true lung cancer incidence, population-level screening rates, or treatment trajectories, which limits causal inference. Additionally, administrative coding of smoking status, obesity, and comorbidities such as COPD and chronic kidney disease is subject to undercoding and documentation bias, which may lead to underestimation of these exposures, particularly in populations with limited healthcare engagement. The cross-sectional design precludes causal interpretation, and residual confounding from unmeasured variables, including cancer stage, treatment type, and insurance status, cannot be excluded. The unexpected finding of lower in-hospital mortality among smokers and obese patients likely reflects the obesity paradox and the healthy smoker effect described in hospitalized populations and should be interpreted cautiously rather than as evidence of a protective biological effect. Additionally, the study period spans 2019-2020, which overlaps with the onset of the COVID-19 pandemic in the United States. Pandemic-related disruptions to hospital admissions, care-seeking behavior, and resource allocation in 2020 may have influenced some of our findings, and this should be considered when interpreting the results. Future research integrating NIS data with cancer registry datasets could provide a more comprehensive picture. The incorporation of survey-weighted multivariable models strengthens the validity of our findings by accounting for confounding demographic, clinical, socioeconomic, and geographic factors.

These interpretations are hypothesis-generating and should be validated in future studies incorporating cancer stage, longitudinal outpatient data, and individual-level measures of screening access and socioeconomic status.

## Conclusions

Lung cancer disparities in the United States are influenced by race, comorbidity burden, socioeconomic status, and geographic region. Interventions aimed at improving access to screening, preventive care, and the equitable distribution of healthcare resources are essential for addressing these inequities. This study suggests that structural and systemic inequities, rather than biological differences alone, are principal drivers of racial and regional disparities in inpatient lung cancer outcomes. These inequities are deeply rooted in longstanding social determinants of health, including residential segregation, unequal access to preventive services, and disparities in insurance coverage, which collectively shape patterns of disease presentation and inpatient burden. Addressing these disparities will require coordinated efforts across healthcare systems, public health agencies, and policymakers. Collectively, these findings call for multilevel interventions, including expanding low-dose CT screening access in underserved communities, investing in culturally tailored preventive care, improving chronic disease management, and addressing upstream social determinants of health, including housing stability, insurance coverage, and transportation barriers. Future research should prioritize longitudinal and registry-based studies that capture cancer stage, treatment trajectories, and long-term outcomes across racial, socioeconomic, and geographic groups to better characterize the full scope of these disparities and evaluate the effectiveness of targeted interventions. Without deliberate structural change, racial and regional disparities in lung cancer morbidity and mortality will persist.
